# “Mosaic” Genes Highlight Forces of Genome Diversity and Adaptation

**DOI:** 10.1371/journal.pbio.0020094

**Published:** 2004-04-13

Microbes are arguably the most adaptable organisms on Earth, inhabiting nearly every crevice of nearly every corner of the globe. Some invade the cavities of a wide variety of insects and other invertebrates while others colonize the skin, blood, eyes, and internal organs of animals. Still others thrive in such inhospitable places as the hydrothermal vents of the ocean floor and the Dry Valleys of Antarctica. These “simple” single-celled organisms have evolved unique molecules and strategies over some 3.5 billion years that suit life on the edge. With the sequenced genomes of nearly 140 microbial species in hand, scientists are gaining valuable insights into the nature of this adaptive diversity.[Fig pbio-0020094-g001]

**Figure pbio-0020094-g001:**
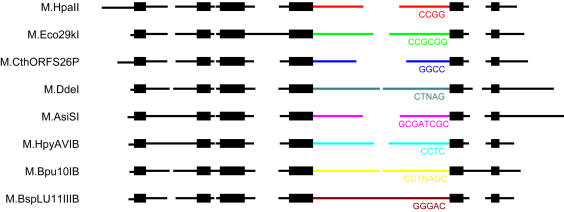
Segmentally variable genes

Adapting to such radically different niches, it appears, has produced genes with diverse functions that evolve at very different rates. Genes that code for molecules essential for fundamental cellular functions like maintaining cell metabolism and structure tend to evolve rather slowly, while genes that make proteins charged with mediating cellular responses to internal or external changes often evolve relatively quickly. Pathogenic microbes in particular rely on a flexible genome to keep a step ahead of their hosts' similarly evolving defenses in the never-ending struggle to gain adaptive advantage. This adaptability underlies the increasing antibiotic resistance of diseases like tuberculosis, as selective pressures favor the expansion of resistant bacterial populations.

Combating such problems requires a molecular understanding of bacterial infections, yet function has been ascribed to only a fraction of the genes found in microbial genomes. One approach to improve functional analyses of genome sequences combines bioinformatics with experimental methods. With such collaborations in mind, Yu Zheng, Richard Roberts, and Simon Kasif have developed a computational approach to help filter out the genetic noise and home in on genomic regions likely to contain clues to gene function. Their method relies on a novel way of classifying genes that flags sequences likely to reward biochemical and genetic efforts to analyze gene function.

Many comparative genomic studies have focused on looking for sequence “motifs” that correlate with well-characterized protein sequences (that is, the amino acid sequence) and predicting function based on their similarity to the known protein sequences. Zheng, Roberts, and Kasif took a different approach, classifying genes based on their sequence variation. The researchers analyzed 43 fully sequenced microbial genomes and, after determining the degree of conservation or divergence among similar genes in different species, divided the genes into three broad categories: rapidly evolving genes unique to a particular species; highly conserved genes; and “segmentally variable,” or mosaic, genes. Stipulating that the boundaries between the categories are somewhat blurred, Zheng et al. define segmentally variable genes as regions that show a mosaic pattern of one or more rapidly evolving, variable regions interspersed with conserved regions. Based on evidence suggesting that retained variable regions tend to serve a function, the researchers predicted that these mosaic genes, with their highly variable, fast-evolving regions, would shed light on the forces that shape genome diversity and adaptation.

For most of the microbes analyzed, mosaic genes accounted for about 8–20% of their genomes. Selecting several large families of mosaic genes, Zheng et al. explored the relationship between genes with known function and the structure of their variable regions. Noting an overabundance of particular functional categories in different species—such as signaling proteins that come into either direct or indirect contact with the cell's environment—the researchers speculate that the variable regions may constitute an adaptive layer for the microbe, as they not only “play a key role in mediating interactions with other molecules” but also support a microbe's ability to adapt to its particular niche. Several bacteria species, for example, contain roughly 40% more mosaic sensor genes involved in cell motility, which the authors attribute to the microbes' “expanded ability to detect different chemical signals and find favorable environments.”

This regional variability appears to reflect the influence of selective pressures that fuel diversity through ongoing interactions with other rapidly evolving molecules in the environment, adding another source of genetic adaptability as cells adjust to new environments and outmaneuver pathogenic threats. While many of the mosaic genes identified encode proteins involved in host-pathogen interactions, defense mechanisms, and intracellular responses to external changes, their function is only broadly understood. While Zheng et al. cannot say to what extent variability affects function—Is extreme variability required for diversity or can modest variation suffice?—they are refining their classification of segmentally variable genes to address such questions. Until then, the authors' “mosaic” approach to understanding gene function promises to improve efforts to annotate the volumes of sequenced genomes on hand, offering biologists a much-needed tool to sift through the mountains of genomic datasets and identify promising targets for further study.

